# Age-period-cohort analysis with a constant-relative-variation constraint for an apportionment of period and cohort slopes

**DOI:** 10.1371/journal.pone.0226678

**Published:** 2019-12-19

**Authors:** Shih-Yung Su, Wen-Chung Lee

**Affiliations:** 1 Institute of Epidemiology and Preventive Medicine, College of Public Health, National Taiwan University, Taipei, Taiwan; 2 Innovation and Policy Center for Population Health and Sustainable Environment, College of Public Health, National Taiwan University, Taipei, Taiwan; 3 Taiwan Cancer Registry, Taipei, Taiwan; Consiglio Nazionale delle Ricerche, ITALY

## Abstract

Age-period-cohort analysis of incidence and/or mortality data has received much attention in the literature. To circumvent the non-identifiability problem inherent in the age-period-cohort model, additional constraints are necessary on the parameters estimates. We propose setting the constraint to reflect the different nature of the three temporal variables: age, period, and birth cohort. There are two assumptions in our method. Recognizing age effects to be deterministic (first assumption), we do not explicitly incorporate the age parameters into constraint. For the stochastic period and cohort effects, we set a constant-relative-variation constraint on their trends (second assumption). The constant-relative-variation constraint dictates that between two stochastic effects, one with a larger curvature gets a larger (absolute) slope, and one with zero curvature gets no slope. We conducted Monte-Carlo simulations to examine the statistical properties of the proposed method and analyzed the data of prostate cancer incidence for whites from 1973–2012 to illustrate the methodology. A driver for the period and/or cohort effect may be lacking in some populations. In that case, the CRV method automatically produces an unbiased age effect and no period and/or cohort effect, thereby addressing the situation properly. However, the method proposed in this paper is not a general purpose model and will produce biased results in many other real-life data scenarios. It is only useful in situations when the age effects are deterministic and dominant, and the period and cohort effects are stochastic and minor.

## Introduction

Age-period-cohort (APC) analysis of disease incidence and mortality rates can provide useful and important information for understanding disease etiology, for evaluating the intervention effect of health policy and medical technology, and for assessing the level of danger of public hazard events [1–6]. The analysis hinges on three temporal variables: age (a person’s age at disease diagnosis, death, or the occurrence of a certain event), period (the calendar year when he/she was diagnosed, died, or when that event occurred), and cohort (his/her year of birth). The literature abounds with APC analyses of various diseases and health conditions.

Because of the exact linear dependency among the three temporal variables, i.e., cohort = period − age, a model with age, period and cohort as the covariates (an APC model) will suffer from the non-identifiability problem; that is, an infinite set of parameter estimates will fit the data equally well, and one cannot single out any one of them from the others. This means that separating age, period and cohort effects is impossible [7–11]. One may attempt to impose additional constraints or assumptions on the APC model in order to obtain a particular set of parameter estimates. However, different sets of constraints often lead to drastically different or even contradictory results. There is no consensus in the APC literature as to which set of constraints is the best and is to be used.

Many researchers urged that the assumptions made by the APC model be justified by theory and stated explicitly [7, 12–14]. In this paper, we acknowledge the different nature of the age, period and cohort effects. Our theory is that age is the internal, biological timing mechanism of an organism, and thereby a person’s disease or mortality rate is a deterministic function of his/her age. For many diseases, we observe that age is also the most important determinant of disease occurrence or death, with the incidence or mortality rate varying hundreds of fold, or more, across the human lifespan. On the other hand, we theorize that period and cohort effects are the manifestations of external, environmental factors. Two examples of drivers of period effects are the implementation of a mass screening program during a specific time period [1, 6, 15] and the gradual improvement of medical care over time [6]. Two examples of drivers of cohort effects are the implementation of a mass hepatitis B vaccination program for newborns born after a certain year, which affects their hepatocellular carcinoma mortality rates in later life [16, 17]; and the exposure to diethylstilbestrol of pregnant women during 1940–1971, which affects clear cell adenocarcinoma and vaginal cancer incidence rates in children born of these pregnancies [18, 19]. (Improvement of medical care may also be a driver of cohort effect if the improvement includes early life care that people carry with it through the life course.) Such outside stimuli can be considered to occur stochastically in time and to perturb the disease rates in a stochastic manner, producing a “stochastic trend” with notable up-and-down variation. (By contrast, a “deterministic trend” should be smoother without too much variation.) We also note that as compared to the deterministic aging process, external factors often exert much weaker effects on incidence/mortality rates. It may even be that an external driver for period and/or cohorts is lacking in some populations. In that case, a plot of disease rate against the calendar year and/or birth year would reveal a flatline—a line without slope and variation.

Recognizing age effects to be deterministic, in this paper we do not explicitly incorporate the age parameters into constraint. For the stochastic period and cohort effects, we set a constraint of constant relative variation (CRV) on the period and cohort slopes. We conduct Monte-Carlo simulations to examine the statistical properties of the proposed method. We analyze data of prostate cancer incidence for whites in the United States to illustrate the methodology. We caution that this is not an APC model for general use. Rather, it has a very specific range of applicability defined by the assumptions imposed on the model. It is only useful in situations when the age effects are deterministic and dominant, and the period and cohort effects are stochastic and minor.

## Materials and methods

### Notations and definitions

Let *y*_*ij*_ denote the occurrence of disease or death cross-classified by age *i* and period *j* (*i* = 1, 2, …, *I* and *j* = 1, 2, …, *J*). We assume the *y*_*ij*_ follow a Poisson distribution with an expectation of *n*_*ij*_ × *r*_*ij*_, where *n*_*ij*_ and *r*_*ij*_ denote the person-year and mortality rate, respectively, for the *i*th age group and the *j*th period group. The APC model is represented by

$$\text{log}\left(r_{ij}\right)=\mu +\alpha_{i}+\beta_{j}+\gamma_{k},$$ (1)

where *μ* is the intercept term, *α*_*i*_, *β*_*j*_, and *γ*_*k*_ are respectively, the age, period, and cohort effects, and *k* (*k* = 1, 2, …, *K*) is the index for the cohort group and is related to *i* and *j* through *k* = *j* − *i* + *I* (the birth year of an individual being equal to his/her year of death minus age at death). Here, the sum-to-zero constraints are used: Σ_*i*_ *α*_*i*_ = Σ_*j*_ *β*_*j*_ = Σ_*k*_ *γ*_*k*_ = 0, or, using matrix notation, **α**^**t**^ **1** = **β**^**t**^ **1 = γ**^**t**^ **1** = 0, where **α** (*I* × 1), **β** (*J* × 1), and **γ** (*K* × 1, *K* = *I* + *J* − 1) are column vectors, with the *i*th, *j*th, and *k*th elements being *α*_*i*_, *β*_*j*_, and *γ*_*k*_, respectively, and the **1**’s are summing vectors of the appropriate dimension with all elements equal to 1.

The age, period, and cohort effects can each be partitioned into a linear slope and a “curvature” component (a term coined by Holford [20] to represent the departures from the linear trend, i.e., the detrended fluctuations). Let the linear slopes for age, period, and cohort effects be denoted by *α*_*L*_, *β*_*L*_, and *γ*_*L*_, respectively (three scalars), and the curvature components, be denoted by **α**_*C*_, **β**_*C*_, and **γ**_*C*_, respectively (three column vectors); that is, $\alpha =\alpha_{L}{\times l}_{\alpha }+\alpha_{C}$, $\beta =\beta_{L}\times l_{\beta }+\beta_{C}$, and $\gamma =\gamma_{L}{\times l}_{\gamma }+\gamma_{C}$, respectively, where $l_{\alpha }$, $l_{\beta }$, and $l_{\gamma }$ are column vectors with the *i*th, *j*th, and *k*th elements being *i* − (*I* + 1)/2, *j* − (*J* + 1)/2, and *k* − (*K* + 1)/2, respectively.

### Representations for the infinite set of parameter estimates of the APC model

Due to the exact linear relationship between age, period, and cohort, the APC model is non-identifiable, meaning that a maximum likelihood estimation (MLE) determines not one, but an infinite set of parameter estimates with the equal goodness of fit. Let ${\hat{\alpha }}^{*}$ (slope: ${\hat{\alpha }}_{L}^{*}$; curvature: ${\hat{\alpha }}_{C}^{*}$,), ${\hat{\beta }}^{*}$ (slope: *${\hat{\beta }}_{L}^{*}$*; curvature: ${\hat{\beta }}_{C}^{*}$,), and ${\hat{\gamma }}^{*}$ (slope: ${\hat{\gamma }}_{L}^{*}$; curvature: ${\hat{\gamma }}_{C}^{*}$,) denote one particular set of parameter estimates. The infinite set of MLEs for the APC model can be represented by

$${\hat{\alpha }}^{\left(u\right)}=\left({\hat{\alpha }}_{L}^{*}+u\right){\times l}_{\alpha }+{\hat{\alpha }}_{C}^{*},$$ (2)

$${\hat{\beta }}^{\left(u\right)}=\left({\hat{\beta }}_{L}^{*}-u\right){\times l}_{\beta }+{\hat{\beta }}_{C}^{*},$$ (3)

and

$${\hat{\gamma }}^{\left(u\right)}=\left({\hat{\gamma }}_{L}^{*}+u\right){\times l}_{\gamma }+{\hat{\gamma }}_{C}^{*},$$ (4)

where *u* is an arbitrary value. Note that the infinite set of MLEs shares the same curvature components (${\hat{\alpha }}_{C}^{*}$, ${\hat{\beta }}_{C}^{*}$ and ${\hat{\gamma }}_{C}^{*}$). The slopes vary (${\hat{\alpha }}_{L}^{\left(u\right)}={\hat{\alpha }}_{L}^{*}+u$, ${\hat{\beta }}_{L}^{\left(u\right)}={\hat{\beta }}_{L}^{*}-u$, and ${\hat{\gamma }}_{L}^{\left(u\right)}={\hat{\gamma }}_{L}^{*}+u$), but two sums (age slope + period slope, *S*^AP^, and period slope + cohort slope, *S*^PC^) are conserved, that is, ${\hat{\alpha }}_{L}^{\left(u\right)}+{\hat{\beta }}_{L}^{\left(u\right)}={\hat{\alpha }}_{L}^{*}+{\hat{\beta }}_{L}^{*}=S^{AP}$ and ${\hat{\beta }}_{L}^{\left(u\right)}+{\hat{\gamma }}_{L}^{\left(u\right)}={\hat{\beta }}_{L}^{*}+{\hat{\gamma }}_{L}^{*}=S^{PC}$.

The infinite set of MLEs for the APC model can alternatively be represented by

$${\hat{\alpha }}^{\left(v\right)}=\left(S^{AP}-v\times S^{PC}\right){\times l}_{\alpha }+{\hat{\alpha }}_{C}^{*},$$ (5)

$${\hat{\beta }}^{\left(v\right)}=\left(v\times S^{PC}\right)\times l_{\beta }+{\hat{\beta }}_{C}^{*},$$ (6)

and

$${\hat{\gamma }}^{\left(v\right)}=\left[\left(1-v\right)\times S^{PC}\right]{\times l}_{\gamma }+{\hat{\gamma }}_{C}^{*},$$ (7)

where v is again an arbitrary value. We will use this latter representation throughout this paper, which involves one arbitrary constant, two conserved slope sums and three identifiable curvature vectors. The reason that we prefer the v parameterization in Eqs (5)–(7) over the *u* parameterization in Eqs (2)–(4) is that v is interpretable if its value is between zero and one; from Eqs (6) and (7), it is clearly the proportion of the slope sum *S*^PC^ that is allocated to the period effects.

### Additional constraints for identification

To uniquely identify the parameters of the APC model, one needs to impose one additional constraint/assumption to the usual sum-to-zero constraints. Let *L*(*μ*, **α**, **β**, **γ**|*y*_*ij*_) denote the likelihood function of the APC model. Fu [21], and Knight and Fu [22] considered a penalized log-likelihood of the form

$$\text{log}\left(L(\mu ,\alpha ,\beta ,\gamma |y_{ij})\right)-\lambda \times \left(\alpha^{t}\alpha +\beta^{t}\beta +\gamma^{t}\gamma \right),$$ (8)

with a tuning parameter *λ* > 0. The penalty term in the parentheses after λ in the likelihood is the sum of the “squared amplitudes” of the age effects (**α**^t^ **α**), period effects (**β**^t^ **β**) and cohort effects (**γ**^t^ **γ**). Intuitively, an introduction of such a term penalizes a model with large age, period and cohort effects. A maximization of the above objective function leads to the so called intrinsic estimators (IEs):

$${\hat{\alpha }}^{IE}=\left(S^{AP}-v^{IE}{\times S}^{PC}\right)\times l_{\alpha }+{\hat{\alpha }}_{C}^{*},$$ (9)

$${\hat{\beta }}^{IE}=\left(v^{IE}\times S^{PC}\right)\times l_{\beta }+{\hat{\beta }}_{C}^{*},$$ (10)

and

$${\hat{\gamma }}^{IE}=\left[\left(1-v^{IE}\right)\times S^{PC}\right]{\times l}_{\gamma }+{\hat{\gamma }}_{C}^{*},$$ (11)

where $v^{IE}=\frac{S^{AP}\times \left({l_{\alpha }}^{t}l_{\alpha }\right)+S^{PC}\times \left({l_{\gamma }}^{t}l_{\gamma }\right)}{S^{PC}\times [\left({l_{\alpha }}^{t}l_{\alpha }\right)+\left({l_{\beta }}^{t}l_{\beta }\right)+\left({l_{\gamma }}^{t}l_{\gamma }\right)]}$. The slopes of the intrinsic estimators satisfy the following constraint:

$${\hat{\alpha }}_{L}^{IE}\times \left({l_{\alpha }}^{t}l_{\alpha }\right)-{\hat{\beta }}_{L}^{IE}\times \left({l_{\beta }}^{t}l_{\beta }\right)+{\hat{\gamma }}_{L}^{IE}\times \left({l_{\gamma }}^{t}l_{\gamma }\right)=0.$$ (12)

Lee and Lin [23] proposed a trend surface (TS) method, with a simpler slope constraint of

$${\hat{\alpha }}_{L}^{TS}-{\hat{\beta }}_{L}^{TS}+{\hat{\gamma }}_{L}^{TS}=0,$$ (13)

and therefore $v^{TS}=\frac{S^{AP}+S^{PC}}{3{\times S}^{PC}}$. It can be shown that this TS constraint corresponds to a maximization of the following penalized log-likelihood:

$$\text{log}\left(L\left(\mu ,\alpha ,\beta ,\gamma \right|y_{ij})\right)-\lambda \times \left(\frac{\alpha^{t}\alpha }{{l_{\alpha }}^{t}l_{\alpha }}+\frac{\beta^{t}\beta }{{l_{\beta }}^{t}l_{\beta }}+\frac{\gamma^{t}\gamma }{{l_{\gamma }}^{t}l_{\gamma }}\right),$$ (14)

again with a tuning parameter *λ* > 0. It can be seen that the penalty term now becomes the sum of the “standardized” squared amplitudes of the three temporal effects, standardizing with respect to the squared amplitudes of the temporal factors *per se* (${l_{\alpha }}^{t}l_{\alpha }$, ${l_{\beta }}^{t}l_{\beta }$ and ${l_{\gamma }}^{t}l_{\gamma }$, respectively).

Tu et al [24, 25] applied the partial least squares (PLS) method for APC analysis. Unfortunately, the results depend on the coding schemes used for the three temporal variables. The PLS method produces the same result as the IE method when the indicator variable (one for true, zero for false) is used for coding whether a data point is in a particular category of a temporal variable, and it produces the same result as the TS method when orthogonal polynomials (the above $l_{\alpha }$, $l_{\beta }$, and $l_{\gamma }$ being the first-order polynomials) are used for coding. It has also been pointed out that the separation of age, period and cohort effects by the IE method by itself depends on the number of age, period and cohort categories [10, 12]. IE method also has a non-uniqueness property that its results show a high variability on different types of dummy parameterization [26].

Osmond and Gardner’s (OG) method [27] hinges on all two-factor models being identifiable. Let ${\hat{\alpha }}^{AP}$ and ${\hat{\beta }}^{AP}$ denote, respectively, the age and period effects of an AP model, i.e., a model with only age and period parameters, or equivalently, an APC model with all the cohort parameters forced to be zero: ${\hat{\gamma }}^{AP}=0$. Similarly, let ${\hat{\alpha }}^{AC}$, ${\hat{\beta }}^{AC}=0$, and ${\hat{\gamma }}^{AC}$ denote the effects for the AC model, and ${\hat{\alpha }}^{PC}=0$, ${\hat{\beta }}^{PC}$, and ${\hat{\gamma }}^{PC}$, the effects for the PC model. The OG method calls for minimizing the weighted sum of the Euclidean distances (in a parameter space with *I* + *J* + *K* dimensions) between the AP, AC, and PC models, respectively, and the full-fledged APC model (parameterized by v, to be consistent in this paper):

$$v^{OG}=argmin\left(\frac{D^{AP}(v)}{{MRSS}^{AP}}+\frac{D^{AC}(v)}{{MRSS}^{AC}}+\frac{D^{PC}(v)}{{MRSS}^{PC}}\right),$$ (15)

where $D^{AP}\left(v\right),$ $D^{AC}\left(v\right)$ and $D^{PC}\left(v\right)$ are the distances to the APC model, and MRSS^AP^, MRSS^AC^ and MRSS^PC^ are the mean residual sums of squares, for the AP, AC and PC models, respectively. Eq (15) above can be viewed as the additional constraint imposed in the OG method. Alternatively, one can derive the OG estimate from a penalized maximum likelihood estimation, with the following penalized log-likelihood:

$$\text{log}\left(L\left(\mu ,\alpha ,\beta ,\gamma \right|y_{ij})\right)-\lambda \times \left(\frac{D^{AP}\left(v\right)}{{MRSS}^{AP}}+\frac{D^{AC}\left(v\right)}{{MRSS}^{AC}}+\frac{D^{PC}\left(v\right)}{{MRSS}^{PC}}\right),$$ (16)

with a tuning parameter *λ* > 0.

Lee and Lin [28] proposed an autoregressive APC model with the cohort effects modeled as a first-order autoregressive process (hereafter referred to as the AR method). The following conditional log-likelihood is to be maximized:

$$\text{log}\left(L\left(\mu ,\alpha ,\beta ,\gamma \right|y_{ij})\right)+\text{log}\left(L(\phi ,\sigma^{2}|\gamma )\right),$$ (17)

where *L*(*ϕ*, *σ*^2^|**γ**) is the likelihood of the autoregressive process (*ϕ*: the autocorrelation, *σ*^2^: the variance, of the stochastic cohort effects). The second term in Eq (17) can be viewed as a constraint for the cohort effects, which will exact a penalty to the overall likelihood if the cohort parameters deviate from the assumed autoregressive process. With such a constraint imposed, the autoregressive APC model is identifiable. The results, though, do not belong to the above solution set parameterized by v.

Clayton and Schifflers [29, 30] (hereafter referred to as the CS method) introduced the age-drift model, which is a model with the age parameters plus a period or a cohort slope. (The fits of the age-plus-period-slope and the age-plus-cohort-slope models to a given dataset are the same, hence the generic term “drift” is used here.) They established a hierarchy of models: (i) the age model (a model with only the age parameters), (ii) the age-drift model, (iii) the AP and AC models, and (iv) the APC model (age + drift + period curvature + cohort curvature), and suggested a logical order (see Fig 2 in reference 30) with sequential statistical tests to find a model with an adequate fit. It has been shown that models with the drift parameter are identifiable even without an additional constraint. However, the question still remains as to how to further partition the somewhat elusive drift into the more tangible, period and cohort slopes, respectively. Chauvel et al [31] proposed the APC-detrended (APCD) and the APC-hysteresis (APCH) models. These models focus specifically on the detrended fluctuations (curvature components) of the cohorts effects and make no attempt to separate the period and cohort slopes.

### The proposed method

Define the “root mean square curvature” (RMSC) for the period effects as

$${\hat{RMSC}}_{\beta }=\sqrt{\frac{{{\hat{\beta }}_{C}^{*}}^{t}{\hat{\beta }}_{C}^{*}}{J}}.$$ (18)

This is a measure of deviation from linearity for the period effects. With ${\hat{RMSC}}_{\beta }=0$(perfect linearity), the slope (change in effects per one unit period) as measured from any two periods is a constant value. While with ${\hat{RMSC}}_{\beta }>0$, the slope no longer remains constant but will vary to a more degree as the index gets larger. Meanwhile, the ${\hat{\beta }}_{L}^{*}$ as introduced earlier can be viewed as an estimate of the expected values of the period slopes, measured from two randomly chosen periods. Therefore, we may calculate the “relative variation” (RV) in period slopes as

$${\hat{RV}}_{\beta }=\frac{{\hat{RMSC}}_{\beta }}{{\hat{\beta }}_{L}^{*}},$$ (19)

a scale-free measure which quantifies the variation of period slopes in relative terms. Similarly, the RMSC and RV for the cohort effects are

$${\hat{RMSC}}_{\gamma }=\sqrt{\frac{{{\hat{\gamma }}_{C}^{*}}^{t}{\hat{\gamma }}_{C}^{*}}{K}}$$ (20)

and

$${\hat{RV}}_{\gamma }=\frac{{\hat{RMSC}}_{\gamma }}{{\hat{\gamma }}_{L}^{*}},$$ (21)

respectively.

As pointed out earlier, first, we assume age effects to be deterministic and period and cohort effects to be stochastic. Therefore, we do not explicitly incoporate the age parameters into constraint. Second, we assume constant relative variation for the period and cohort slopes. Our constraint is, therefore:

$${\hat{RV}}_{\beta }={\hat{RV}}_{\gamma }.$$ (22)

With this CRV constraint imposed, the APC model is identifiable with the v parameter being (see S1 Appendix)

$$v^{CRV}=\frac{{\hat{RMSC}}_{\beta }}{{\hat{RMSC}}_{\beta }+{\hat{RMSC}}_{\gamma }}.$$ (23)

The CRV estimates can be found using the $v^{CRV}$:

$${\hat{\alpha }}^{CRV}=\left(S^{AP}-v^{CRV}{\times S}^{PC}\right)\times l_{\alpha }+{\hat{\alpha }}_{C}^{*},$$ (24)

$${\hat{\beta }}^{CRV}=\left(v^{CRV}\times S^{PC}\right)\times l_{\beta }+{\hat{\beta }}_{C}^{*},$$ (25)

and

$${\hat{\gamma }}^{CRV}=[\left(1-v^{CRV}\right)\times S^{PC}]{\times l}_{\gamma }+{\hat{\gamma }}_{C}^{*}.$$ (26)

Here we see that a simple CRV constraint for the period and cohort slopes [Eq (22)] will affect the estimates for all three temporal effects in Eqs (24), (25) and (26), through $v^{CRV}$ in Eq (23).

Because ${\hat{RMSC}}_{\beta }\geq 0$ and ${\hat{RMSC}}_{\gamma }\geq 0$, $v^{CRV}$ in Eq (23) is guaranteed to be between zero and one (we let $v^{CRV}=0.5$, if ${\hat{RMSC}}_{\beta }={\hat{RMSC}}_{\gamma }=0$). Therefore, $v^{CRV}$ is readily recognized to be the proportion of the total period and cohort slopes (*S*^PC^) allocated to the period effects, and ${(1-v}^{CRV})$, that allocated to the cohort effects. From Eq (23), we also see that the apportionment by the CRV method is determined according to the magnitudes of the slope variation (as measured by the root mean square curvatures), of the period and the cohort effects, respectively. The CRV constraint dictates that between the two stochastic effects, one with a larger variation gets a larger (absolute) slope, and one with zero variation gets no slope. The latter property should prove useful in that if any effect is zero, i.e. a flatline without variation, the CRV constraint will guarantee that its slope is zero.

The CRV constraint can also be derived from a maximization of the following penalized log-likelihood:

$$\text{log}\left(L(\mu ,\alpha ,\beta ,\gamma |y_{ij})\right)-\lambda \times \left(\frac{\beta^{t}\beta }{{RMSC}_{\beta }\times \left({l_{\beta }}^{t}l_{\beta }\right)}+\frac{\gamma^{t}\gamma }{{RMSC}_{\gamma }\times \left({l_{\gamma }}^{t}l_{\gamma }\right)}\right),$$ (27)

with a tuning parameter *λ* > 0 (in S2 Appendix). It is of interest to compare the penalty term of the CRV method in Eqation (27) with those of the IE and TS methods in Eqs (8) and (14), respectively. First, we see that unlike the IE and TS methods, the CRV penalty does not involve the **α**^t^ **α** term (the age effects are still affected by this CRV penalty as previously mentioned). Second, we see that the penalties imposed on the period and cohort parameters are proportional to the standardized squared amplitudes ($\frac{\beta^{t}\beta }{{l_{\beta }}^{t}l_{\beta }}$ and $\frac{\gamma^{t}\gamma }{{l_{\gamma }}^{t}l_{\gamma }}$, respectively) (as in the TS method), and additionally, inversely proportional to the mean square curvatures (RMSC_*β*_ and RMSC_*γ*_, respectively) just introduced.

To find the CRV estimate, one begins with an arbitrary APC estimate: ${\hat{\alpha }}^{*}$, ${\hat{\beta }}^{*}$ and ${\hat{\gamma }}^{*}$. (These can be obtained by setting any arbitrary constraint: say, *β*_1_ = *β*_2_). For this estimate, one extracts the slopes (${\hat{\alpha }}_{L}^{*},{\hat{\beta }}_{L}^{*},{\hat{\gamma }}_{L}^{*}$) and curvatures (${\hat{\alpha }}_{C}^{*},{\hat{\beta }}_{C}^{*},{\hat{\gamma }}_{C}^{*}$), using the simple formulas presented in S3 Appendix. One then uses Eqs (18) and (20) to calculate ${\hat{RMSC}}_{\beta }$ and ${\hat{RMSC}}_{\gamma }$, and Eq (23) to calculate $v^{CRV}$. Finally, one uses Eqs (24)–(26) to calculate the CRV estimate.

Eq (23) is the key to apportion period and cohort slopes in the proposed CRV method. To check the robustness of the apportionment, one can re-compute a $v^{CRV}$ based on the root mean square curvatures of the older periods and cohorts, and one based on those of the recent periods and cohorts, and compare the results with the original $v^{CRV}$. If the three $v^{CRV}’s$ differ too much, the CRV method should not be used.

### Simulation setups

We simulate a population with age-period cross-classified mortality data containing a total of nine age groups: 40–44, 45–49, …, 80–84, and a total of eight period groups: 1976–1980, 1981–1985, …, 2011–2015. We set up a population of one million with the same age distribution as the year 2000 World Health Organization standard population. The population size and the age distribution are set up to be stable over time. Because all APC methods (including IE, TS, OG, AR and CRV) were estimated using the aggregated-level data, the sample size for each simulation data is 72 (the total number of cell from the age-period table).

We set up an APC model [Eq (1)] for the mortality rates and let the death counts follow a Poisson distribution. The intercept of the APC model is set up to be *μ* = −5, which corresponds to ≈674 deaths per 100 000 person-year. The age effects (*α*_*i*_, for *i* = 1, 2, …, 9) are set up to be *α*_1_ = −2.35, *α*_2_ = −1.45, *α*_3_ = −0.93, *α*_4_ = −0.35, *α*_5_ = −0.04, *α*_6_ = −0.32, *α*_7_ = −0.98, *α*_8_ = −1.48, *α*_9_ = −2.26, respectively (denoted as A in the simulation study). The rate ratio between 80–84 and 40–44 year-old people is exp(*α*_9_ − *α*_1_) ≈ 100, representing a typical age effect for cancer mortality.

The period and cohort effects are by contrast assumed to be stochastic, and therefore, their assumed values can be different in different rounds of the simulation. We design two mechanisms (P_I_ and P_II_) to generate the stochastic period effects and another two mechanisms (C_I_ and C_II_) to generate the stochastic cohort effects (P_I_ and C_I_ simulate pulse impacts and P_II_ and C_II_ simulate wave impacts, each with a random amplitude and a random starting time, as detailed in S4 Appendix). We also consider the situations when the period effects and/or the cohort effects are absolutely zero (P_zero_ and C_zero_, respectively). We generate data for all nine combinations of mechanisms in turn: A×(P_zero_, P_I_, P_II_)×(C_zero_, C_I_, C_II_).

In addition, we designed seven scenarios specifically to challenge the proposed CRV method: (i) all three temporal effects are absolutely zero (A_zero_×P_zero_×C_zero_), (ii) all three temporal effects are stochastic, (iii) all three temporal effects are deterministic: the setup A for the age effects, a flat but highly variable period effect, and a monotonic linear cohort effect, (iv) all three temporal effects are deterministic: the setup A for the age effects, a monotonic and decreasing linear period effect, and a monotonic and increasing linear cohort effect, (v) all three temporal effects are deterministic: an age effect with a less than 10-fold change in rate between the oldest and the youngest age groups, plus J-shape period and cohort effects, (vi) stochastic period and cohort effects but the CRV assumption fails: RV_*β*_ is far greater than RV_*γ*_, and (vii) stochastic period and cohort effects but the CRV assumption fails: RV_*β*_ is far lower than RV_*γ*_ (details of these additional simulations are described in S5 Appendix).

We use the proposed CRV method to analyze the data. For comparison, we also present the results of the IE, TS, OG, AR, and CS methods. (We did not perform the PLS method, as the results would be the same as those of the IE or TS method depending on the coding used, as explained earlier.) We perform a total of 100 000 simulations for each scenario. The biases of the age, period and cohort effects were calculated as the mean differences between the estimated values and the corresponding true values in the simulation (mean of ${\hat{\alpha }}_{i}-\alpha_{i}$, ${\hat{\beta }}_{j}-\beta_{j}$ and ${\hat{\gamma }}_{k}-\gamma_{k}$, respectively, for age, period and cohort effects for each simulated scenario). The Monte-Carlo standard errors were also calculated and were presented in supporting information (from S1 to S5 Tables).

### Prostate cancer incidence rates in the United States from 1973–2012

As an example, we analyze the data of prostate cancer incidence for whites in the United States from 1973–2012. Data came from the Surveillance, Epidemiology, and End Results (SEER) Program Research Data [32], which includes cancer incidence cases and population in the United States associated by age, sex, race (white, black, American Indian, Asian, Hispanic, non-Hispanic white, etc), years of diagnosis, and geographic areas (following the SEER-9 registry and county). We selected all prostate cancer in white and formed an age-period cross-classified table with 9 age groups (40–44, 45–49, …, 80–84) and 8 period groups (1973–1977, 1978–1982,…, 2008–2012), spanning a total of 16 birth-cohort groups (mid-cohort years: 1893,1898, …, 1968). The sample size (number of cells from 5-year age and period table) is 72. The age and calendar year of population were similarly categorized.

## Results

### Simulation results

**Fig 1:**
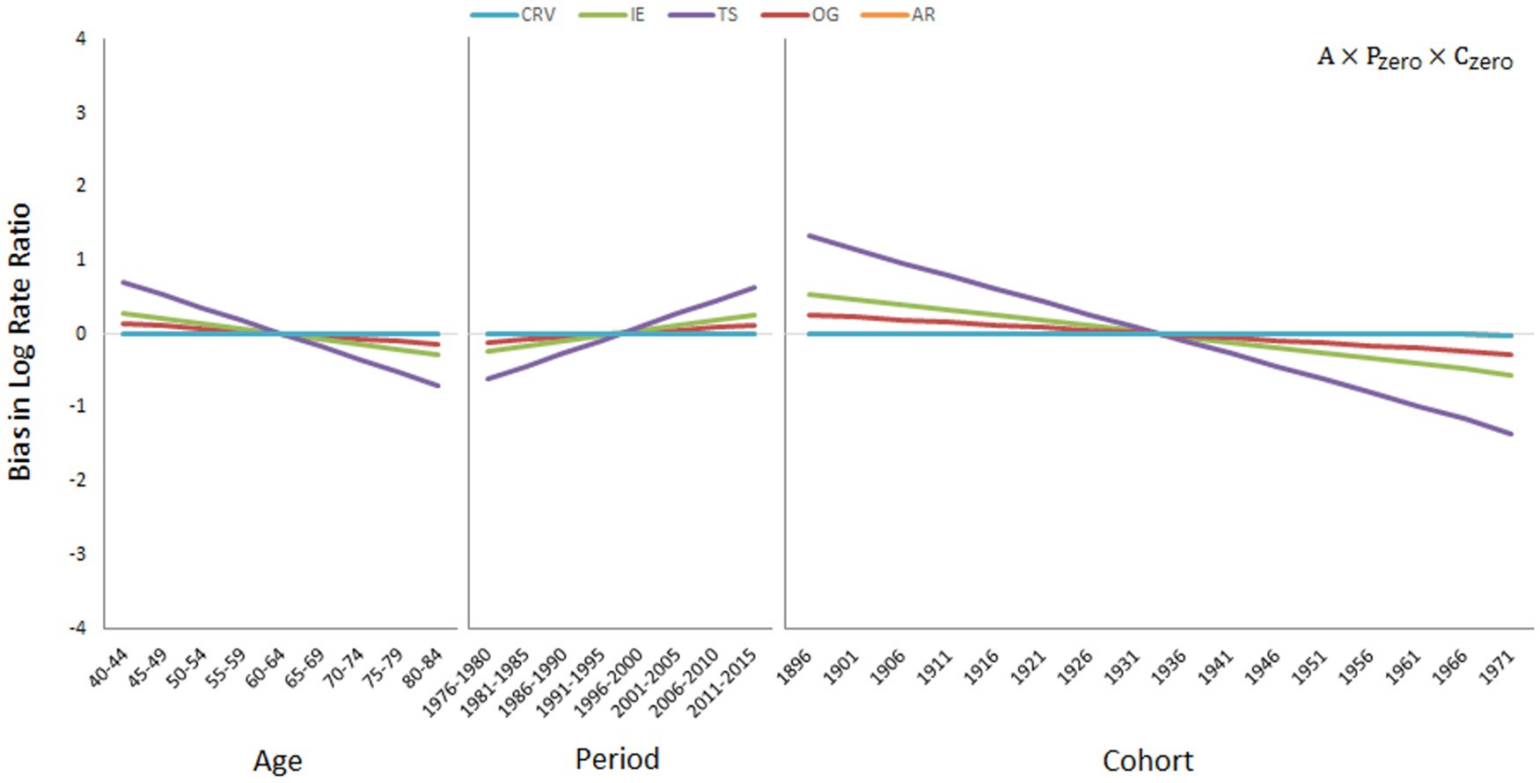
Simulation results when only the deterministic age effect is present (A×P_zero_×C_zero_).

Fig 1 presents the simulation results when only the deterministic age effect is present (A×P_zero_×C_zero_). The CRV and the AR methods are approximately unbiased. By contrast, the IE, OG, and TS methods are seriously biased. For the age effect, the three biased methods overestimate the age effect for the young and underestimate it for the elderly. For the period and cohort effects that are actually zero, these methods produce positive period slopes of 0.03 (OG), 0.07 (IE), and 0.17 (TS) per five calendar years, and negative cohort slopes of -0.03 (OG), -0.07 (IE), and -0.17 (TS) per five birth-cohort years. The biases are a simple linear function of age, period, and cohort variables, that is, the three methods are biased in estimating the slopes but not the curvature components. This is because as mentioned previously, methods that admit an *u* or v parameterization share the same curvature components (which are asymptotically unbiased, a property of MLEs), but produce different slope estimates.

**Fig 2:**
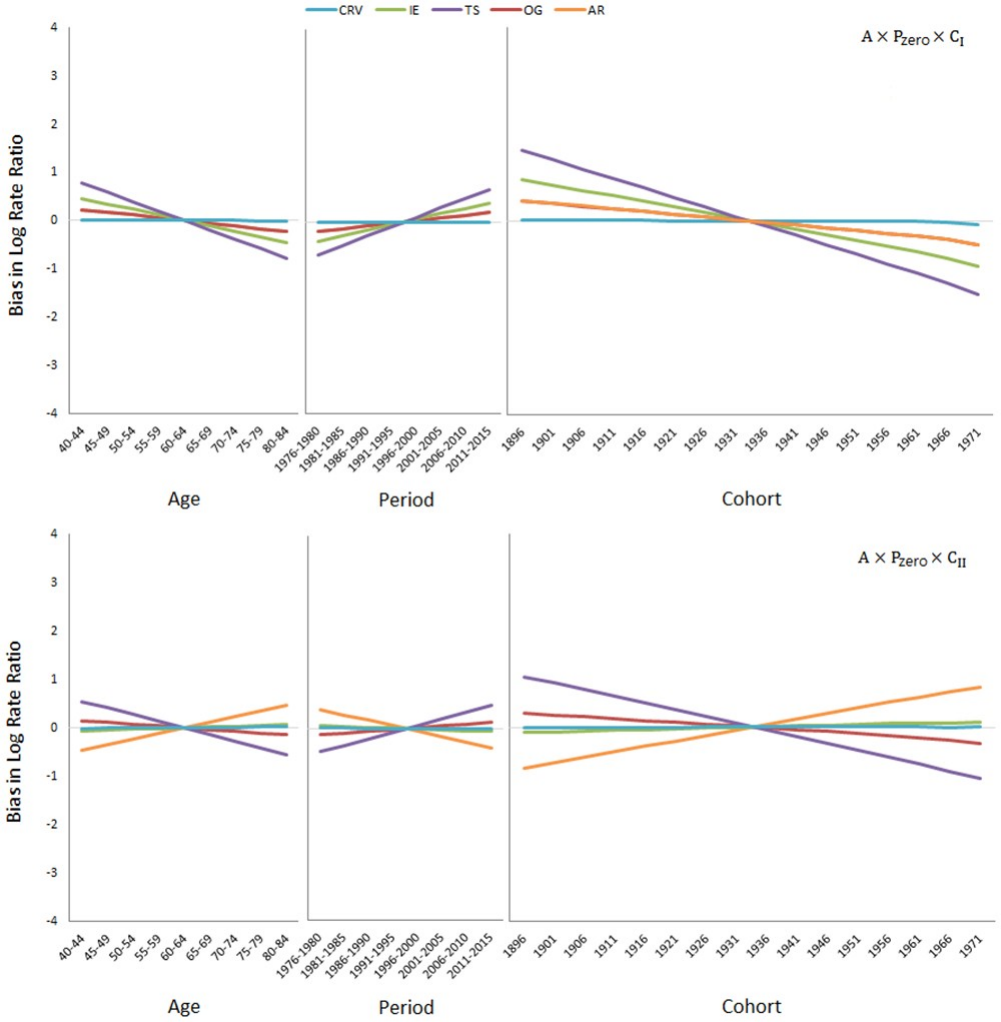
Simulation results when the deterministic age effect and the stochastic cohort effect are present (upper panel: A×P_zero_×C_I_; lower panel: A×P_zero_×C_II_).

**Fig 3:**
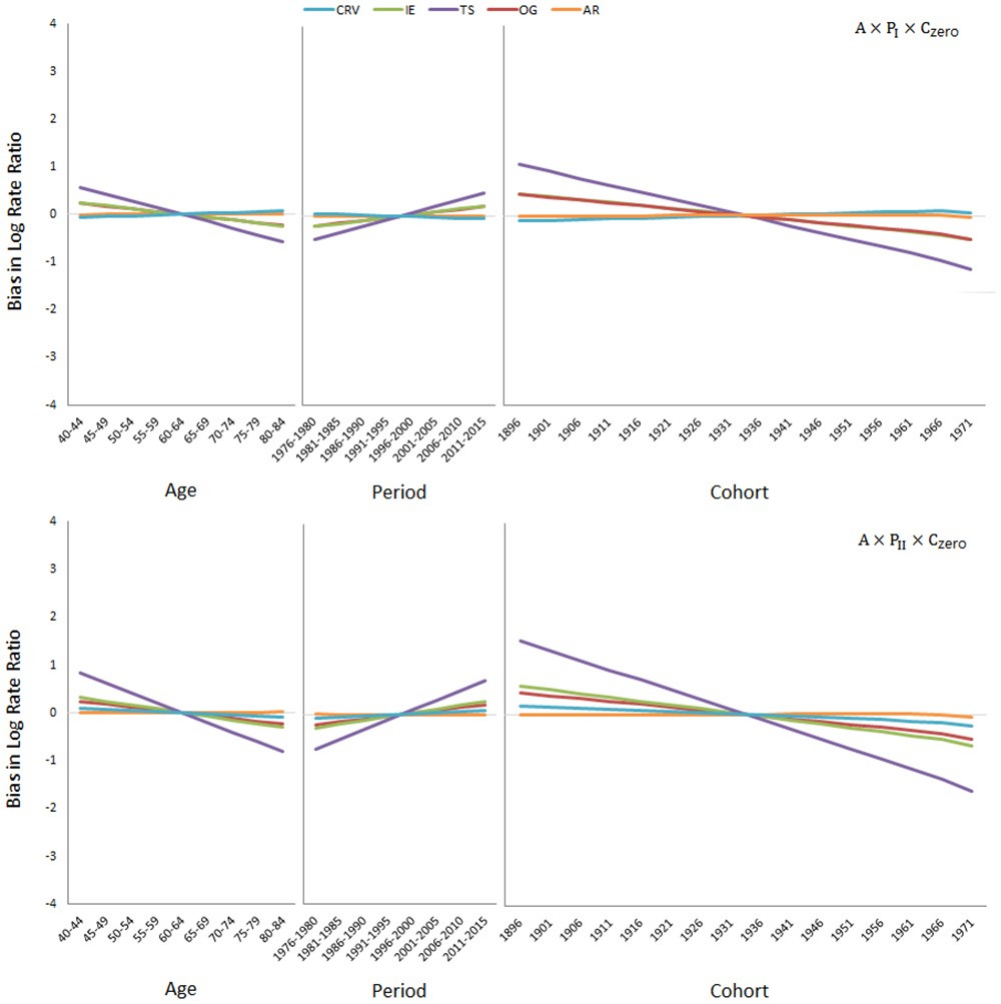
Simulation results when the deterministic age effect and the stochastic period effect are present (upper panel: A×P_I_×C_zero_; lower panel: A×P_II_×C_zero_).

Fig 2 presents the simulation results when in addition to the deterministic age effect, the stochastic cohort effect is also present (upper panel: A×P_zero_×C_I_; lower panel: A×P_zero_×C_II_). The CRV method is again approximately unbiased, but the AR method is now biased; it erroneously reports a positive slope of 0.06 and a negative slope of -0.12 for the period effect per five calendar years for the A×P_zero_×C_I_ and A×P_zero_×C_II_ data, respectively. The other three methods are also biased; they report period slopes of 0.06 (OG), 0.12 (IE), and 0.20 (TS) per five calendar years for the A×P_zero_×C_I_ data, and -0.02 (IE), 0.04 (OG), and 0.14 (TS) per five calendar years for the A×P_zero_×C_II_ data. Fig 3 presents the simulation results when the deterministic age effect and the stochastic period effect are both present (upper panel: A×P_I_×C_zero_; lower panel: A×P_II_×C_zero_). Now, the CRV and AR methods are approximately unbiased, but the IE, OG, and TS methods are severely biased.

**Fig 4:**
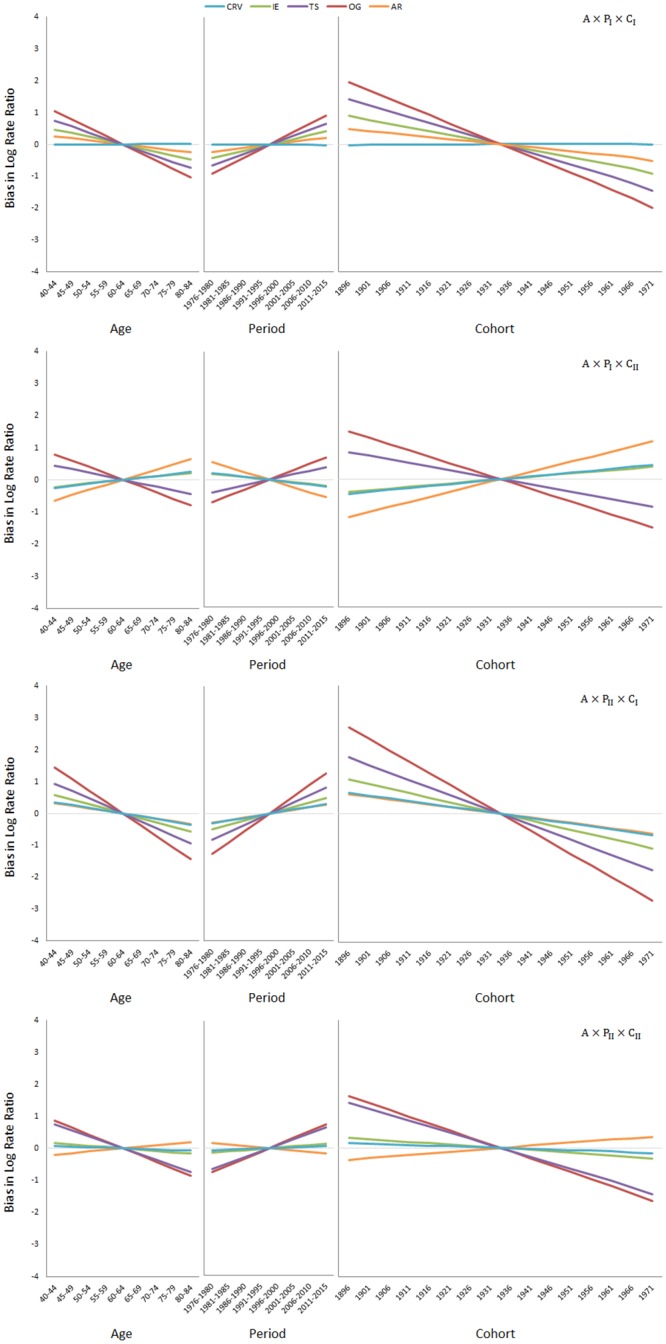
Simulation results when the deterministic age effect, and the stochastic period and cohort effects, are present (1st panel: A×P_I_×C_I_; 2nd panel: A×P_I_×C_II_; 3rd panel: A×P_II_×C_I_; 4th panel: A×P_II_×C_II_).

Fig 4 presents the simulation results when all three temporal effects are present (1st panel: A×P_I_×C_I_; second panel: A×P_I_×C_II_; third panel: A×P_II_×C_I_; fourth panel: A×P_II_×C_II_). For the A×P_I_×C_I_ data, the CRV method is approximately unbiased and the other four methods are severely biased (AR, IE, TS, and OG, in ascending order of the magnitude of bias). For the A×P_I_×C_II_ data, the CRV and IE methods are slightly biased and the other three methods are seriously biased. For the A×P_II_×C_I_ data, all methods are biased to some extent, but among them, the CRV and AR methods are the least biased. For the A×P_II_×C_II_ data, the CRV method is again approximately unbiased. The other four methods are biased to various degrees: IE (slightly biased), AR (moderately biased), TS and OG (severely biased).

**Fig 5:**
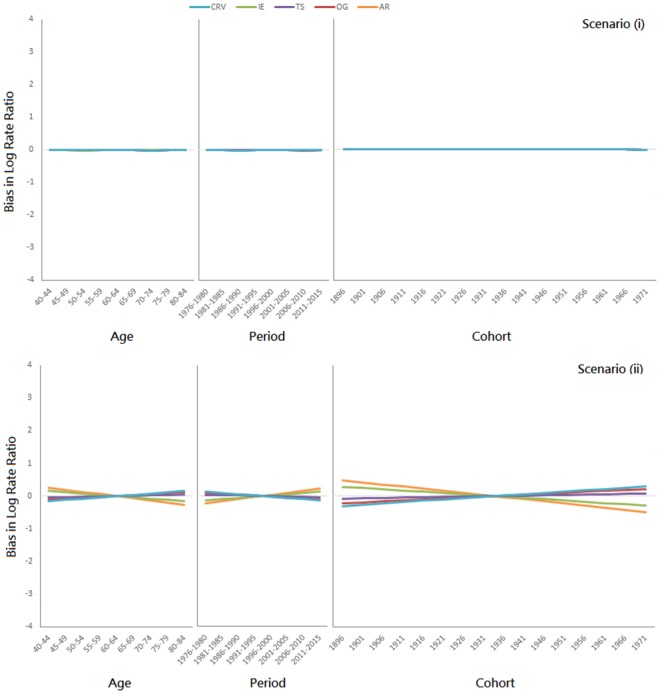
Additional simulation results when all three temporal effects are zero (1st panel: Scenario (i)) and when all three temporal effects are stochastic (2nd panel: Scenario (ii)).

**Fig 6:**
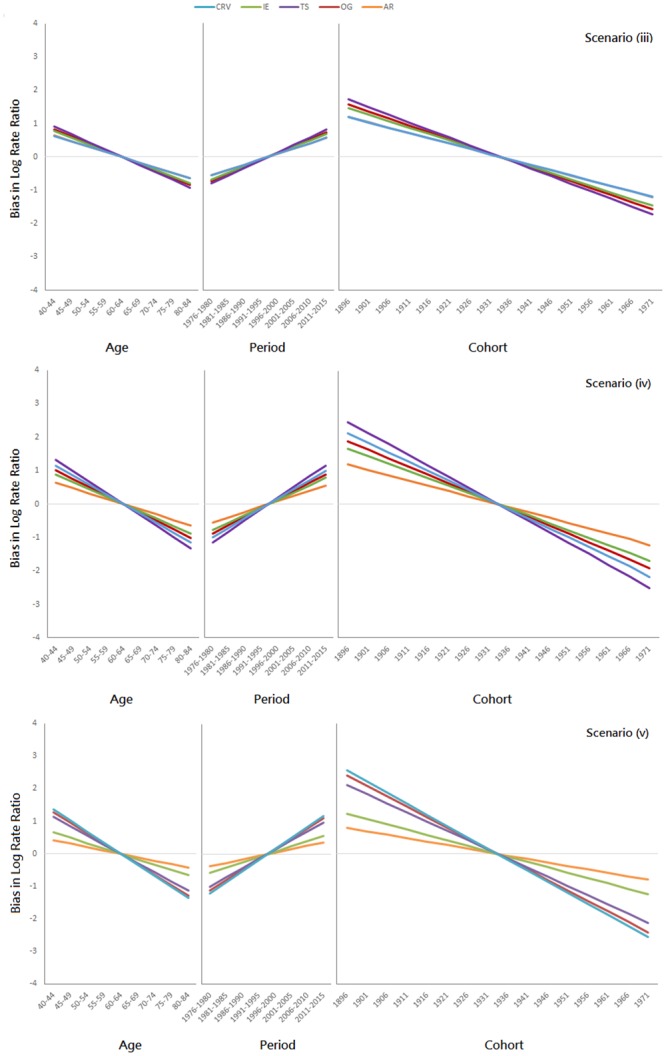
Additional simulation results when all three temporal effects are deterministic (1st panel: Scenario (iii); 2nd panel: Scenario (iv); 3rd panel: Scenario(v)).

**Fig 7:**
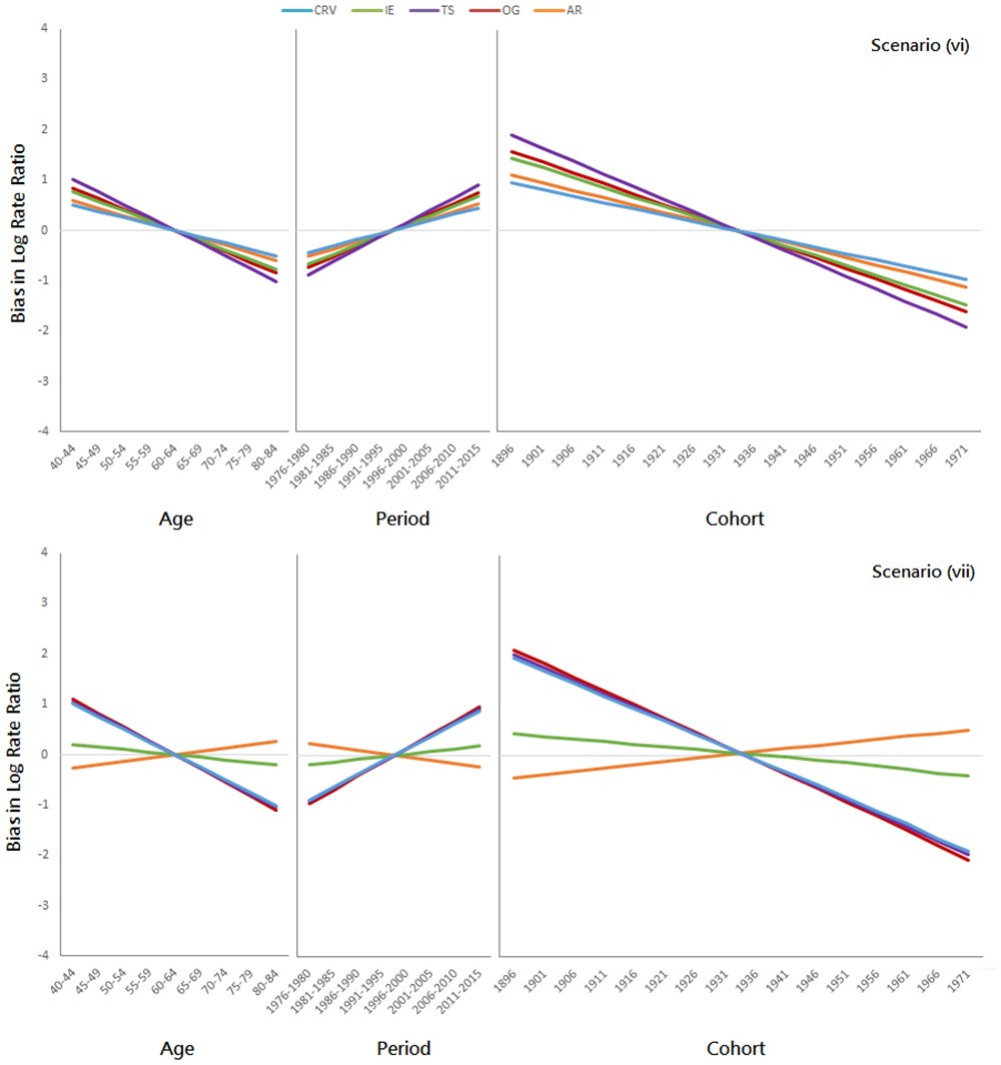
Additional simulation results when the CRV assumption fails (1st panel: Scenario (vi): RV_*β*_ is far greater than RV_γ_; 2nd panel: Scenario (vii): RV_*β*_ is far lower than RV_*γ*_).

Results for the additional simulations (i, ii, …, vii) are presented in Figs 5, 6 and 7, respectively. When all three temporal effects are absolutely zero (i), all five methods are unbiased (Fig 5, 1st panel). When all three temporal effects are stochastic (ii), all five methods are no more than slightly biased (Fig 5, 2nd panel). When all three temporal effects are deterministic (iii, iv, v), all five methods are biased (Fig 6); all methods overestimate the age effect for the young and underestimate it for the elderly, underestimate the period effect for the earlier periods and overestimate it for the later ones, and overestimate the cohort effect for the older cohorts and underestimate it for the recent ones. When the CRV assumption fails, all five methods are biased (Fig 7). The directions of the biases are consistent with the results in Fig 6, except for the AR method.

### Example results

**Fig 8:**
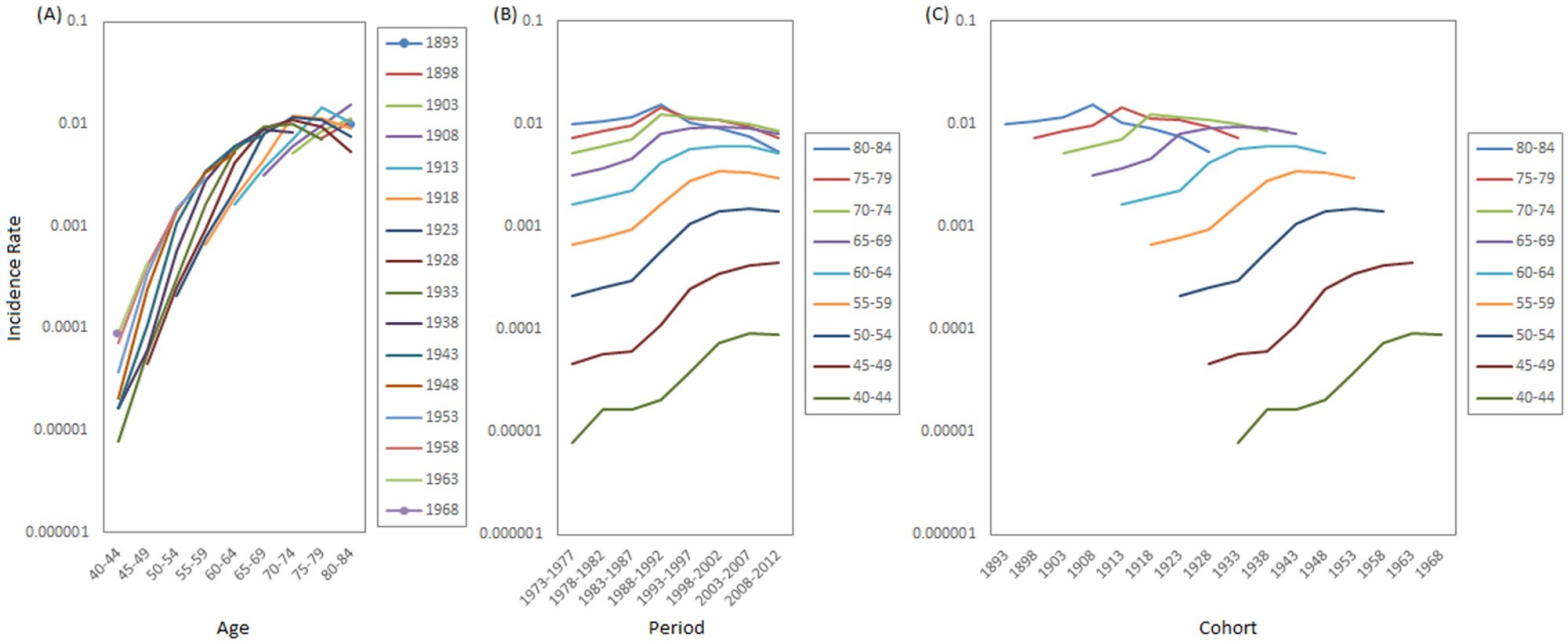
Prostate cancer incidence for whites in the United States from 1973–2012 (A: Age curves for different birth cohorts; B: Secular trends for different ages; C: Birth-cohort trends for different ages).

A simple graphical depiction of the prostate cancer data is presented in Fig 8. The prostate cancer incidence rate of the oldest age group of 80–84 is ≈1200 times that of the youngest age group of 40–44 (Fig 8A). The prostate cancer incidence shows inconsistent long-term period trends in different age groups (Fig 8B). Incidence rates increase steeply for the younger age groups but increase gently and then level off for the older age groups. And for the oldest age group of 80–84, an outright decreasing long-term trend is noted instead. Superimposed in these disparate long-term trends are two short-term trends that are more or less consistent across age groups: a brief increase in the early periods, and a brief decrease in the later ones, respectively. By contrast, the birth-cohort trends are more consistent across age groups (Fig 8C). For the earliest few birth cohorts, the trends are to slightly decrease and then to level off. For the later birth cohorts, the trends are an initial slight increase followed by a drastic increase for the most recent ones. S6 Table presents the prostate cancer incidence rates (per 100,000) in whites by age and period groups.

**Fig 9:**
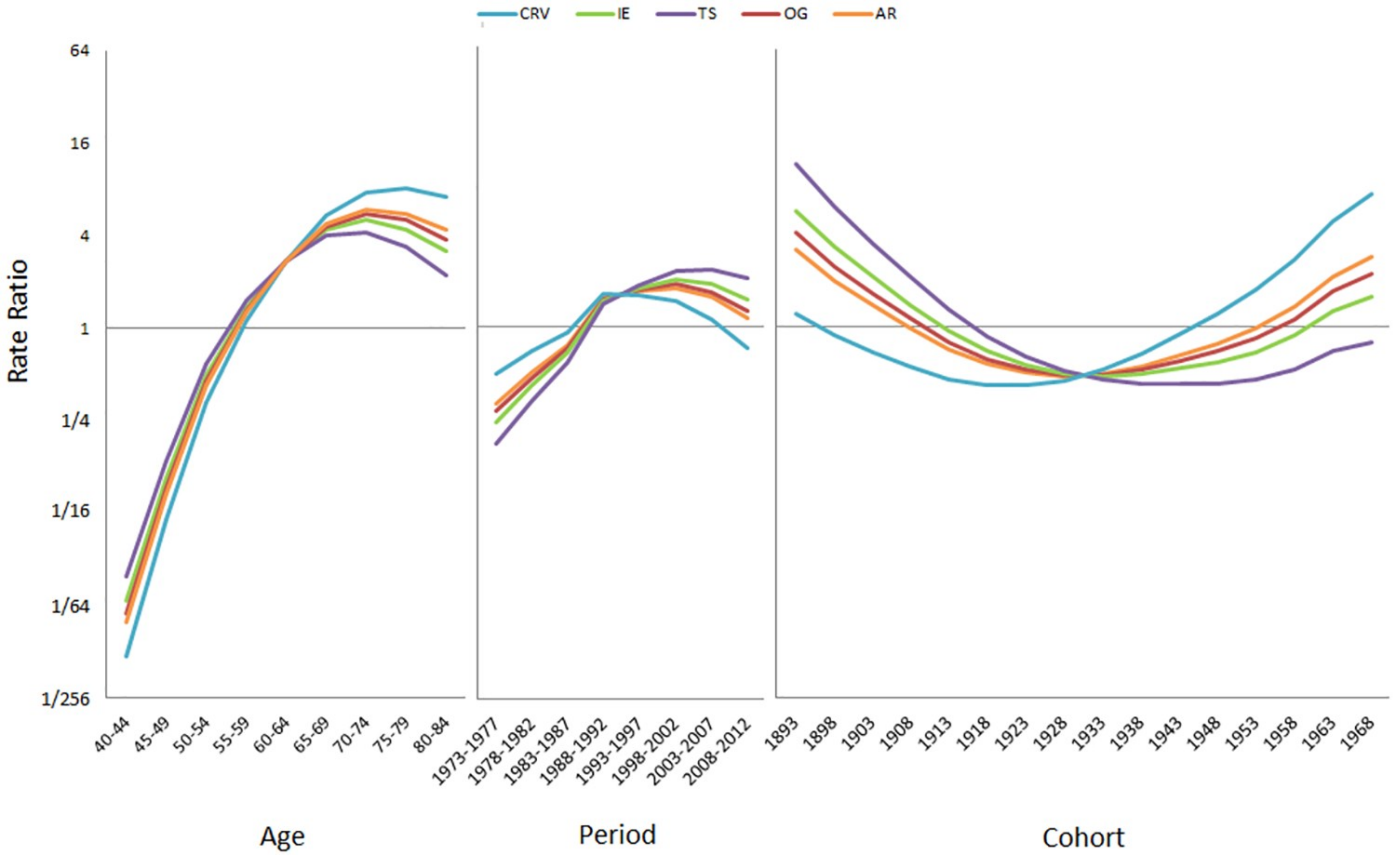
Results of age-period-cohort analysis for the prostate cancer data using various methods (CRV: The proposed method of constant relative variation; IE: Method of intrinsic estimators; TS: Trend surface method; OG: Osmond and Gardner’s method; AR: Autoregressive model).

The results of APC modeling are presented in Table 1 and Fig 9. Even without an additional constraint, two slope sums can be estimated, and these are 0.92 (the sum of the age and period slopes) and 0.20 (the sum of the period and cohort slopes), respectively (Table 1). The CRV method allocates approximately one-third ($v^{CRV}=0.37$) of the latter sum (0.20) to be the period slope (0.08), and the remaining two-thirds, the cohort slope (0.13). This then leaves 0.84 for the age slope. The apportionment of the slopes is insensitive to the calculation of the root mean square curvature using different data ranges: $v^{CRV}=0.37$ when using older periods (1973–1992) and cohorts (1893–1948) and $v^{CRV}=0.39$ when using recent periods (1993–2012) and cohorts (1913–1968).

**Table 1:** Parameter estimates and bootstrap standard errors of age-period-cohort analysis for the prostate cancer data using various methods.

	CRV		IE		TS		OG		AR	
	Estimate	SE	Estimate	SE	Estimate	SE	Estimate	SE	Estimate	SE
Age slope	0.8398	0.0022	0.6347	0.0021	0.5428	0.0016	0.6795	0.0022	0.7202	0.0038
Period slope	0.0768	0.0005	0.2819	0.0017	0.3738	0.0015	0.2371	0.0015	0.1959	0.0043
Cohort slope	0.1279	0.0016	-0.0772	0.0003	-0.1691	0.0009	-0.0324	0.0010	0.0083	0.0027
Sum of the age and period slopes	0.9166		0.9166		0.9166		0.9166		0.9161	
Sum of the period and cohort slopes	0.2047		0.2047		0.2047		0.2047		0.2042	

CRV: the proposed method of constant relative variation; IE: method of intrinsic estimators; TS: trend surface method; OG: Osmond and Gardner’s method; AR: autoregressive model.

In Fig 9, we see that the CRV method reports a ≈1000-fold increase in incidence rate from the youngest age group to the oldest, a mild 3.3-fold increase/2.2-fold decrease in the incidence rate in the early/late periods, and a mild 3-fold decrease in incidence rate from 1893 to 1918 birth cohorts, followed by a conspicuous 18-fold increase in the rate all the way to the most recent 1968 birth cohort. These results are largely in line with the previous graphical analysis (Fig 8). The CRV estimates and the corresponding bootstrapped standard errors were presented in S7 Table.

Prostate cancer is predominantly a cancer of the elderly, having the steepest age curve among all major cancers in men [33–35]. Cancer registries in many countries/regions around the globe observe the same tremendous increase of a thousand fold in prostate cancer incidence from ages 40–44 to ages 80–84 as we did in this study [33, 36, 37]. The period effect derived from our CRV method for prostate cancer incidence in the United States is consistent with previous studies [38–41], that is, an increasing trend since 1973, a peak at 1992, and a declining trend thereafter. The researchers of previous studies have attributed such a period effect to the practices of prostate cancer screening in the United States. Specifically, the initial segment of rising incidence may be due to the increased detection of tumors resulting from increased prostate-specific antigen (PSA) screening since the mid-1980s [40]. However, the trend in the rate of first-time PSA procedures started to decline after 1992 [38], leading to the decline of prostate cancer incidence [41]. As for the birth-cohort effect, we note that men born between 1908 and 1928 have the lowest prostate cancer risks. These are the people who experienced World War I (1917–1918), the Great Depression (1929–1939), or World War II (1941–1945) in their early childhoods. Prostate cancer is an affluent type of cancer [42]. Higher intakes of red meat, saturated fat and dairy products are associated with higher prostate cancer risks [43]. This may help explain why the risk of prostate cancer increases dramatically for men born well after those lean years.

The results of the four other APC methods are also presented in the same table/fig for comparison. They yield exactly the same (or nearly so as in the case of AR) slope sums as the CRV method (Table 1). However, they disagree on how these slope sums should be further divided into the three temporal factors. In Fig 9, we see that for the age effect, they report much smaller increases in rate, 360-fold (AR), 270-fold (OG), 190-fold (IE) and 90-fold (TS), respectively, from the youngest age group to the oldest. For the period effect, they all report a long-term increasing trend, 3.6-fold (AR), 4.5-fold (OG), 6.1-fold (IE) and 12-fold (TS) increases in rate, respectively, from 1973–1977 to 2008–2012. For the cohort effect, they report a major decrease in incidence rate (AR: 6.8-fold; OG: 8.4-fold; IE: 12-fold; TS: 25-fold) from the 1893 to the 1933 birth-cohort followed by a minor increase in rate (AR: 5.7-fold; OG: 4.6-fold; IE: 3.3-fold; TS: 1.8-fold) to the 1968 birth-cohort. As for the CS method, it determines a full-fledged APC model, but the non-identifiability problem remains.

For the purpose of comparison, we also conducted APC analysis for prostate cancer incidence in Taiwan from 1979–2013 (S6 Appendix, S8 Table, S1 and S2 Figs). The sample size (number of cells in S8 Table) is 63. The age effects of prostate cancer in Taiwan using the CRV method were similar to those in the United States. The period effects and cohort trends in Taiwan, however, were both continuously increasing (PSA tesing rate remains very low in Taiwan as compared to the United States). The results of the four other APC methods (in the same S2 Fig) in Taiwan reported smaller age effects and larger period effects. The cohort effects in TS, IE and AR methods reported a decreasing or flat trend which is contrary to that found in the graphical analysis (S1 Fig).

## Discussion

The proposed CRV method is based on setting a constraint of constant relative variation on the period and cohort slopes [Eq (22)]. The CRV constraint can also be derived from a maximization of a penalized log-likelihood function [Eq (27)], with the parameter governing the penalization approaching zero. So in the limit, there is no or a very minimal constraint imposed by the method. The CRV result also belongs to the class of so-called “perpendicular solutions” [44], being perpendicular to the following null vector: $\left(0^{t},-\frac{{l_{\beta }}^{t}}{{\hat{RMSC}}_{\beta }\times \left({l_{\beta }}^{t}l_{\beta }\right)},\frac{{l_{\gamma }}^{t}}{{\hat{RMSC}}_{\gamma }\times \left({l_{\gamma }}^{t}l_{\gamma }\right)}\right)$. A recent study analyzed the statistical properties of the IE method [45]. By comparison, the proposed CRV method is rather naïve, and its statistical properties need to be further investigated using the same mathematical rigor.

A driver for the period and/or cohort effect may be lacking in some populations. In that case, the CRV method automatically produces an unbiased age effect and no period and/or cohort effect, thereby addressing the situation properly (see Figs 1, 2 and 3). None of the other methods, IE, TS, OG or AR, shares this desirable property. The method of Carstensen [46] can partly achieve this. If for example, the period effect is known *a priori* to be non-existent or to play only a minor role, *as per* Carstensen’s method, one can run an age-cohort model first and then use the residual terms to fit a period model. The result is indeed period effects with a small slope or no slope at all. However, to use Carstensen’s method, one needs to know beforehand which effect is lacking. By comparison, one simply lets the data speak for themselves in the CRV method.

There are two assumptions in our proposed method. We assume deterministic age effects and stochastic period and cohort effects. For conditions other than diseases and mortalities, age may not necessarily be the most important determinant for temporal trends and, therefore, to qualify for a special do-not-constrain status as in our method. For example, a number of studies have indicated that human social behavior is heavily influenced by the external and social environment [47, 48]. By contrast, age effects are less remarkable; less than a 10-fold change in rates were observed between the oldest and the youngest age groups in studies regarding drinking behavior, religious service and activity, social capital and trust, marijuana consumption, and social inequality, among others [49–54]. Our method will certainly fail in this situation (Scenario (v) in Fig 6). It is also possible that period and/or cohort effects by themselves are also deterministic, such as monotonic/near-linear period and/or cohort trend as a result of medical process, or they may be stochastic but do not satisfy constant relative variation (our second assumption), such as a smooth but conspicuous linear trend in one and a flat but highly variable trend in the other. Our method will fail again in these scenarios (Scenarios (iii) and (iv) in Fig 6; Scenarios (vi) and (vii) in Fig 7).

In conclusion, the method proposed in this paper is not an APC model for general use. It is only useful in situations when the age effects are deterministic and dominant, and the period and cohort effects are stochastic and minor.

## Supporting information

S1 Appendix Derivation from Eqs (22) to (23). (DOCX)

S2 Appendix Deriving the CRV constraint from a maximization of the penalized log-likelihood. (DOCX)

S3 Appendix Formulas for extracting the slopes and curvatures from an arbitrary APC solution. (DOCX)

S4 Appendix Mechanisms for generating the stochastic period and cohort effects. (DOCX)

S5 Appendix Additional simulation setups. (DOCX)

S6 Appendix Data source of prostate cancer incidence rates in Taiwan from 1979–2013. (DOCX)

S1 Fig Prostate cancer incidence in Taiwan from 1979–2013 (A: Age curves for different birth cohorts; B: Secular trends for different ages; C: Birth-cohort trends for different ages). (TIF)

S2 Fig Results of age-period-cohort analysis for the prostate cancer data in Taiwan using various methods. (TIF)

S1 Table Monte-Carlo standard error for CRV estimates. (DOCX)

S2 Table Monte-Carlo standard error for IE estimates. (DOCX)

S3 Table Monte-Carlo standard error for TS estimates. (DOCX)

S4 Table Monte-Carlo standard error for OG estimates. (DOCX)

S5 Table Monte-Carlo standard error for AR estimates. (DOCX)

S6 Table Prostate cancer incidence rates (per 100,000) in whites by age and period groups. (DOCX)

S7 Table The bootstrapped standard error of CRV estimates. (DOCX)

S8 Table The prostate cancer incidence rates (per 100,000) in Taiwan by age and period groups. (DOCX)
