# Supplementary material for: Age-period-cohort analysis with a constant-relative-variation constraint for an apportionment of period and cohort slopes
Source: PLoS One. 2019 Dec 19;14(12):e0226678. doi: 10.1371/journal.pone.0226678 (PMC6922428; doi:10.1371/journal.pone.0226678)
Supplement: S1 Appendix — (DOCX) [file pone.0226678.s001.docx]

**S1 Appendix.** **Derivation from equation (22) to equation (23)**

The proposed constraint [equation (22)] is

$$\frac{{\hat{\mathrm{RMSC}}}_{\beta}}{\hat{\beta}_{L}^{*}}\boldsymbol{=}\frac{{\hat{\mathrm{RMSC}}}_{\gamma}}{\hat{\gamma}_{L}^{*}}\mathbf{,}$$

and $\hat{\beta}_{L}^{*}$ is

$$\hat{\beta}_{L}^{*}=\hat{\gamma}_{L}^{*}\times\frac{{\hat{\mathrm{RMSC}}}_{\beta}}{{\hat{\mathrm{RMSC}}}_{\gamma}}\mathbf{.}$$

Because the sum of period and cohort slopes ($S^{\mathrm{PC}}=\hat{\gamma}_{L}^{*}+\hat{\beta}_{L}^{*}$) is conserved for the infinite set of MLEs for the APC model, we can find that:

$$S^{\mathrm{PC}}=\hat{\gamma}_{L}^{*}\times\left( 1+\hat{\gamma}_{L}^{*}\frac{{\hat{\mathrm{RMSC}}}_{\beta}}{{\hat{\mathrm{RMSC}}}_{\gamma}} \right)$$

$$\frac{{\hat{\mathrm{RMSC}}}_{\gamma}}{{\hat{\mathrm{RMSC}}}_{\beta}+{\hat{\mathrm{RMSC}}}_{\gamma}}\times S^{\mathrm{PC}}=\hat{\gamma}_{L}^{*}$$

$$\left( 1\mathbf{-}\frac{{\hat{\mathrm{RMSC}}}_{\beta}}{{\hat{\mathrm{RMSC}}}_{\beta}+{\hat{\mathrm{RMSC}}}_{\gamma}} \right)\times S^{\mathrm{PC}}=\hat{\gamma}_{L}^{*}$$

where the $\mathcal{v}$ parameter is

$$\mathcal{v=}\frac{{\hat{\mathrm{RMSC}}}_{\beta}}{{\hat{\mathrm{RMSC}}}_{\beta}+{\hat{\mathrm{RMSC}}}_{\gamma}}$$

as in equation (23).
