# Supplementary material for: Age-period-cohort analysis with a constant-relative-variation constraint for an apportionment of period and cohort slopes
Source: PLoS One. 2019 Dec 19;14(12):e0226678. doi: 10.1371/journal.pone.0226678 (PMC6922428; doi:10.1371/journal.pone.0226678)
Supplement: S2 Appendix — (DOCX) [file pone.0226678.s002.docx]

**S2 Appendix. Deriving the CRV constraint from a maximization of the penalized log-likelihood.**

As mentioned in the text, the infinite set of MLEs for the APC model can be represented by

${\hat{\boldsymbol{\alpha}}}^{(\mathcal{v})}=\left( S^{\mathrm{AP}}-\mathcal{v}\times S^{\mathrm{PC}} \right){\times\mathcal{l}}_{\alpha}+{\hat{\boldsymbol{\alpha}}}_{C}^{*}$,

$${\hat{\boldsymbol{\beta}}}^{(\mathcal{v})}=\left( \mathcal{v}\times S^{\mathrm{PC}} \right)\times\mathcal{l}_{\beta}+{\hat{\boldsymbol{\beta}}}_{C}^{*},$$

and

$${\hat{\boldsymbol{\gamma}}}^{(\mathcal{v})}=\left[ \left( 1-\mathcal{v} \right)\times S^{\mathrm{PC}} \right]{\times\mathcal{l}}_{\gamma}+{\hat{\boldsymbol{\gamma}}}_{C}^{*},$$

where $\mathcal{v}$ is an arbitrary value. For all $\mathcal{v\in}R$, the log-likelihoods of the APC model,

$\log\left( L\left( \mu\boldsymbol{,}{\hat{\boldsymbol{\alpha}}}^{(\mathcal{v})}\boldsymbol{,}{\hat{\boldsymbol{\beta}}}^{(\mathcal{v})}\boldsymbol{,}{\hat{\boldsymbol{\gamma}}}^{(\mathcal{v})} | y_{ij} \right) \right)$,

are the same. The penalized term,

$\left( \frac{{{\hat{\boldsymbol{\beta}}}^{(\mathcal{v})}}^{t}{\hat{\boldsymbol{\beta}}}^{(\mathcal{v})}}{{\hat{\mathrm{RMSC}}}_{\beta}\times\left( {\mathcal{l}_{\beta}}^{t}\mathcal{l}_{\beta} \right)}\boldsymbol{+}\frac{{{\hat{\boldsymbol{\gamma}}}^{(\mathcal{v})}}^{t}{\hat{\boldsymbol{\gamma}}}^{(\mathcal{v})}}{{\hat{\mathrm{RMSC}}}_{\gamma}\times\left( {\mathcal{l}_{\gamma}}^{t}\mathcal{l}_{\gamma} \right)} \right)$,

however, will be minimized when

$\mathcal{v=}\mathcal{v}^{\mathrm{CRV}}=\frac{{\hat{\mathrm{RMSC}}}_{\beta}}{{\hat{\mathrm{RMSC}}}_{\beta}+{\hat{\mathrm{RMSC}}}_{\gamma}}$.

For any $\left( {\hat{\boldsymbol{\alpha}}}^{*}\boldsymbol{,}{\hat{\boldsymbol{\beta}}}^{*}\boldsymbol{,}{\hat{\boldsymbol{\gamma}}}^{*} \right)\notin\left\{ \left( {\hat{\boldsymbol{\alpha}}}^{(\mathcal{v})}\boldsymbol{,}{\hat{\boldsymbol{\beta}}}^{(\mathcal{v})}\boldsymbol{,}{\hat{\boldsymbol{\gamma}}}^{(\mathcal{v})} \right): \mathcal{v\in}R \right\}$, its log-likelihood of the APC model is smaller than that of $\left( {\hat{\boldsymbol{\alpha}}}^{\mathrm{CRV}}\boldsymbol{,}{\hat{\boldsymbol{\beta}}}^{\mathrm{CRV}}\boldsymbol{,}{\hat{\boldsymbol{\gamma}}}^{\mathrm{CRV}} \right)$, but the penalized term may also be smaller than the corresponding term of $\left( {\hat{\boldsymbol{\alpha}}}^{\mathrm{CRV}}\boldsymbol{,}{\hat{\boldsymbol{\beta}}}^{\mathrm{CRV}}\boldsymbol{,}{\hat{\boldsymbol{\gamma}}}^{\mathrm{CRV}} \right)$. However, when $\lambda\to0^{+}$, the APC log-likelihood term dominates. Therefore, we see that $\mathcal{v=}\mathcal{v}^{\mathrm{CRV}}$ maximizes the penalized log-likelihood

$\log\left( L\left( \mu\boldsymbol{,}\boldsymbol{\alpha}\boldsymbol{,}\boldsymbol{\beta}\boldsymbol{,}\boldsymbol{\gamma} | y_{ij} \right) \right)-\lambda\times\left( \frac{\boldsymbol{\beta}^{t}\boldsymbol{\beta}}{\mathrm{RMSC}_{\beta}\times\left( {\mathcal{l}_{\beta}}^{t}\mathcal{l}_{\beta} \right)}\boldsymbol{+}\frac{\boldsymbol{\gamma}^{t}\boldsymbol{\gamma}}{\mathrm{RMSC}_{\gamma}\times\left( {\mathcal{l}_{\gamma}}^{t}\mathcal{l}_{\gamma} \right)} \right)$,

with $\lambda\to0^{+}$.
