# Supplementary material for: Age-period-cohort analysis with a constant-relative-variation constraint for an apportionment of period and cohort slopes
Source: PLoS One. 2019 Dec 19;14(12):e0226678. doi: 10.1371/journal.pone.0226678 (PMC6922428; doi:10.1371/journal.pone.0226678)
Supplement: S3 Appendix — (DOCX) [file pone.0226678.s003.docx]

**S3 Appendix. Formulas for extracting the slopes and curvatures from an arbitrary APC solution.**

Let (${\hat{\boldsymbol{\alpha}}}^{*}$, ${\hat{\boldsymbol{\beta}}}^{*}$ and ${\hat{\boldsymbol{\gamma}}}^{*}$) denote an arbitrary APC solution, which complies with the sum-to-zero constraints:${{\hat{\boldsymbol{\alpha}}}^{*}}^{t}\mathbf{1}={{\hat{\boldsymbol{\beta}}}^{*}}^{t}\mathbf{1}={{\hat{\boldsymbol{\gamma}}}^{*}}^{t}\mathbf{1}=0$. The slopes are

$\hat{\alpha}_{L}^{*}=\frac{{\mathcal{l}_{\alpha}}^{t}{\hat{\boldsymbol{\alpha}}}^{*}}{{\mathcal{l}_{\alpha}}^{t}\mathcal{l}_{\alpha}}$,

$\hat{\beta}_{L}^{*}=\frac{{\mathcal{l}_{\beta}}^{t}{\hat{\boldsymbol{\beta}}}^{*}}{{\mathcal{l}_{\beta}}^{t}\mathcal{l}_{\beta}}$,

and

$\hat{\gamma}_{L}^{*}=\frac{{\mathcal{l}_{\gamma}}^{t}{\hat{\boldsymbol{\gamma}}}^{*}}{{\mathcal{l}_{\gamma}}^{t}\mathcal{l}_{\gamma}}$,

respectively. The curvatures are

${\hat{\boldsymbol{\alpha}}}_{C}^{*}={\hat{\boldsymbol{\alpha}}}^{*}-\hat{\alpha}_{L}^{*}{\times\mathcal{l}}_{\alpha}$*,*

${\hat{\boldsymbol{\beta}}}_{C}^{*}={\hat{\boldsymbol{\beta}}}^{*}-{\hat{\beta}_{L}^{*}\times\mathcal{l}}_{\beta}$,

and

${\hat{\boldsymbol{\gamma}}}_{C}^{*}={\hat{\boldsymbol{\gamma}}}^{*}-{\hat{\gamma}_{L}^{*}\times\mathcal{l}}_{\gamma}$,

respectively.
