# Supplementary material for: Age-period-cohort analysis with a constant-relative-variation constraint for an apportionment of period and cohort slopes
Source: PLoS One. 2019 Dec 19;14(12):e0226678. doi: 10.1371/journal.pone.0226678 (PMC6922428; doi:10.1371/journal.pone.0226678)
Supplement: S4 Appendix — (DOCX) [file pone.0226678.s004.docx]

**S4 Appendix. Mechanisms for generating the stochastic period and cohort effects.**

For stochastic period effects, we first generate

$$\boldsymbol{\beta}^{I}=a\times\max\left\{ \sin\left( \frac{2\pi}{J}\times\boldsymbol{t} \right),0 \right\}$$

and

$\boldsymbol{\beta}^{\mathrm{II}}=a\times\max\left\{ \sin\left( \frac{2\pi}{J}\times\boldsymbol{t} \right),0 \right\}+b\times\min\left\{ \sin\left( \frac{2\pi}{J}\times\boldsymbol{t} \right),0 \right\}$,

where

$\boldsymbol{t}=\max\left\{ \left( \mathcal{l}_{\beta}+\frac{J+1}{2} \right)-s,0 \right\}$,

and the $a$, $b$ (amplitude) and the $s$ (starting time) are the random samples from the uniform$\left( 0, 2 \right)$ distribution and the set $\left\{ 1,2,\ldots, J-1 \right\}$, respectively. Next, we apply the sum-to-zero constraints on $\boldsymbol{\beta}^{I}$ and $\boldsymbol{\beta}^{\mathrm{II}}$ to obtain the $P_{I}$ and $P_{\mathrm{II}}$ effects, respectively.

For stochastic cohort effects, we first generate

$$\boldsymbol{\gamma}^{I}=a\times\max\left\{ \sin\left( \frac{2\pi}{K}\times\boldsymbol{t} \right),0 \right\}$$

and

$\boldsymbol{\gamma}^{\mathrm{II}}=a\times\max\left\{ \sin\left( \frac{2\pi}{K}\times\boldsymbol{t} \right),0 \right\}+b\times\min\left\{ \sin\left( \frac{2\pi}{K}\times\boldsymbol{t} \right),0 \right\}$,

where

$\boldsymbol{t}=\max\left\{ \left( \mathcal{l}_{\gamma}+\frac{K+1}{2} \right)-s,0 \right\}$,

and the $a$, $b$ and the $s$ are the random samples from the uniform$\left( 0, 2 \right)$ distribution and the set $\left\{ 1,2,\ldots, K-1 \right\}$, respectively. Next, we apply the sum-to-zero constraints on $\boldsymbol{\gamma}^{I}$ and $\boldsymbol{\gamma}^{\mathrm{II}}$ to obtain the $C_{I}$ and $C_{\mathrm{II}}$ effects, respectively.
