# Supplementary material for: Age-period-cohort analysis with a constant-relative-variation constraint for an apportionment of period and cohort slopes
Source: PLoS One. 2019 Dec 19;14(12):e0226678. doi: 10.1371/journal.pone.0226678 (PMC6922428; doi:10.1371/journal.pone.0226678)
Supplement: S5 Appendix — (DOCX) [file pone.0226678.s005.docx]

**S5 Appendix. Additional simulation setups**

For stochastic age effect in additional simulation (ii), we first generate

$\boldsymbol{\alpha}^{S}=a\times\max\left\{ \sin\left( \frac{2\pi}{I}\times\boldsymbol{t} \right),0 \right\}$,

where

$\boldsymbol{t}=\max\left\{ \left( \mathcal{l}_{\alpha}+\frac{I+1}{2} \right)-s,0 \right\}$,

and the $a$ and the $s$ are the random samples from the uniform$\left( 0, 3 \right)$ distribution and the set $\left\{ 1,2,\ldots,I-1 \right\}$, respectively. Next, we apply the sum-to-zero constraints on $\boldsymbol{\alpha}^{S}$ to obtain the stochastic age effect, respectively.

For deterministic period and cohort effects in additional simulation (iii), the period effects ($\beta_{j}$, for $j=$1, 2,..., 8) are assumed to be $\beta_{1}=-6.23$, $\beta_{2}=6.25$, $\beta_{3}=0.98$, $\beta_{4}=-0.39$, $\beta_{5}=-0.92$, $\beta_{6}=0.38$, $\beta_{7}=-0.7$, $\beta_{8}=0.65$, respectively, and the cohort effects ($\gamma_{k}$, for $k=$1, 2,..., 16) are assumed to be $\gamma_{1}=-1.2$, $\gamma_{2}=-1.04$, $\gamma_{3}=-0.88$, $\gamma_{4}=-0.72$, $\gamma_{5}=-0.56$, $\gamma_{6}=-0.4$, $\gamma_{7}=-0.24$, $\gamma_{8}=-0.08$, $\gamma_{9}=0.08$, $\gamma_{10}=0.24$, $\gamma_{11}=0.4$, $\gamma_{12}=0.56$, $\gamma_{13}=0.72$, $\gamma_{14}=0.88$, $\gamma_{15}=1.04$, $\gamma_{16}=1.2$, respectively (equal to $\mathcal{l}_{\gamma}\times0.16$).

For deterministic period effects in additional simulation (iv), the period effects ($\beta_{j}$, for $j=$1, 2,..., 8) are assumed to be $\beta_{1}=1.05$, $\beta_{2}=0.75$, $\beta_{3}=0.45$, $\beta_{4}=0.15$, $\beta_{5}=-0.15$, $\beta_{6}=-0.45$, $\beta_{7}=-0.75$, $\beta_{8}=-1.05$, respectively (equal to $-\mathcal{l}_{\beta}\times0.3$).

For deterministic age, period and cohort effects in additional simulation (v), the age effects ($\alpha_{i}$, for $i=$1, 2,..., 9) are assumed to be $\alpha_{1}=-1.28$, $\alpha_{2}=-0.9$, $\alpha_{3}=-0.72$, $\alpha_{4}=-0.39$, $\alpha_{5}=0.1$, $\alpha_{6}=0.37$, $\alpha_{7}=0.8$, $\alpha_{8}=0.99$, $\alpha_{9}=1.03$, respectively, the period effects ($\beta_{j}$, for $j=$1, 2,..., 8) are assumed to be $\beta_{1}=2.01$, $\beta_{2}=1.17$, $\beta_{3}=0.57$, $\beta_{4}=-0.23$, $\beta_{5}=-0.73$, $\beta_{6}=-0.93$, $\beta_{7}=-0.93$, $\beta_{8}=-0.93$, respectively, and the cohort effects ($\gamma_{k}$, for $k=$1, 2,..., 16) are assumed to be $\gamma_{1}=0.57$, $\gamma_{2}=0.02$, $\gamma_{3}=-0.33$, $\gamma_{4}=-0.63$, $\gamma_{5}=-0.83$, $\gamma_{6}=-0.93$, $\gamma_{7}=-0.88$, $\gamma_{8}=-0.78$, $\gamma_{9}=-0.63$, $\gamma_{10}=-0.33$, $\gamma_{11}=-0.08$, $\gamma_{12}=0.32$, $\gamma_{13}=0.62$, $\gamma_{14}=1.02$, $\gamma_{15}=1.32$, $\gamma_{16}=1.57$, respectively.

For stochastic cohort effects in additional simulation (vi), we first generate

$$\boldsymbol{\gamma}^{\mathrm{III}}=a\times0.25\times\max\left\{ \sin\left( \frac{2\pi}{K}\times\boldsymbol{t} \right),0 \right\}+b\times0.25\times\min\left\{ \sin\left( \frac{2\pi}{K}\times\boldsymbol{t} \right),0 \right\}+\left( \mathcal{l}_{\gamma}\times0.16 \right),$$

where

$\boldsymbol{t}=\max\left\{ \left( \mathcal{l}_{\gamma}+\frac{K+1}{2} \right)-s,0 \right\}$,

and the $a$, $b$ and the $s$ are the random samples from the uniform$\left( 0, 1 \right)$ distribution and the set $\left\{ 1,2,\ldots, K-1 \right\}$, respectively. Next, we apply the sum-to-zero constraints on $\boldsymbol{\gamma}^{\mathrm{III}}$ to obtain the additional stochastic cohort effects, respectively.

For stochastic period effects in additional simulation (vii), we first generate

$$\boldsymbol{\beta}^{\mathrm{III}}=a\times0.25\times\max\left\{ \sin\left( \frac{2\pi}{J}\times\boldsymbol{t} \right),0 \right\}+b\times0.25\times\min\left\{ \sin\left( \frac{2\pi}{J}\times\boldsymbol{t} \right),0 \right\}-\left( \mathcal{l}_{\beta}\times0.3 \right),$$

where

$\boldsymbol{t}=\max\left\{ \left( \mathcal{l}_{\beta}+\frac{J+1}{2} \right)-s,0 \right\}$,

and the $a$, $b$ and the $s$ are the random samples from the uniform$\left( 0, 1 \right)$ distribution and the set $\left\{ 1,2,\ldots, J-1 \right\}$, respectively. Next, we apply the sum-to-zero constraints on $\boldsymbol{\beta}^{\mathrm{III}}$ to obtain the additional stochastic period effects, respectively.
