# Supplementary material for: Age-period-cohort analysis with a constant-relative-variation constraint for an apportionment of period and cohort slopes
Source: PLoS One. 2019 Dec 19;14(12):e0226678. doi: 10.1371/journal.pone.0226678 (PMC6922428; doi:10.1371/journal.pone.0226678)
Supplement: S6 Appendix — (DOCX) [file pone.0226678.s006.docx]

**S6 Appendix.** **Data source of prostate cancer incidence rates in Taiwan from 1979-2013.**

Another example of prostate cancer incidence data in Taiwan from 1979-2013 were abstracted from the database provided by the Taiwan Cancer Registry. The age of prostate cancer patients were categorized into nine groups (40-44, 45-49, …, 80-84). Data from patients younger than 40 and older than 85 years old were not used as same as the selection in example of the United States. The calendar years were categorized into 7 groups (1979-1983, 1984-1988,…, 2009-2013). Population numbers from 1979-2013 were abstracted from the database provided by the Department of Statistics of the Ministry of the Interior in Taiwan. The age and calendar year of the population were similarly categorized.
