# Supplementary figures and images for: Age-period-cohort analysis with a constant-relative-variation constraint for an apportionment of period and cohort slopes
Source: PLoS One. 2019 Dec 19;14(12):e0226678. doi: 10.1371/journal.pone.0226678 (PMC6922428; doi:10.1371/journal.pone.0226678)

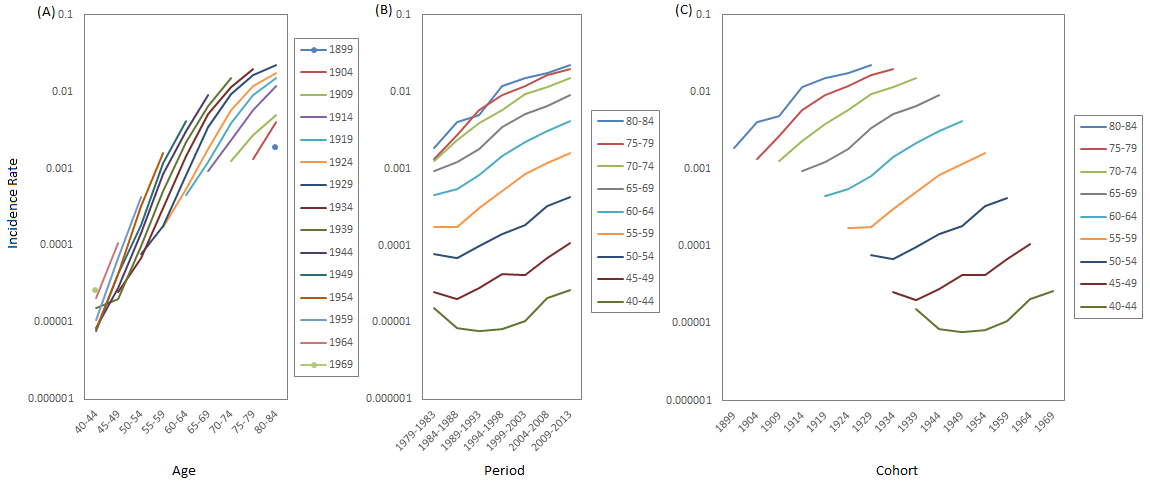

Supplement: S1 Fig — (TIF) [file pone.0226678.s007.tif]

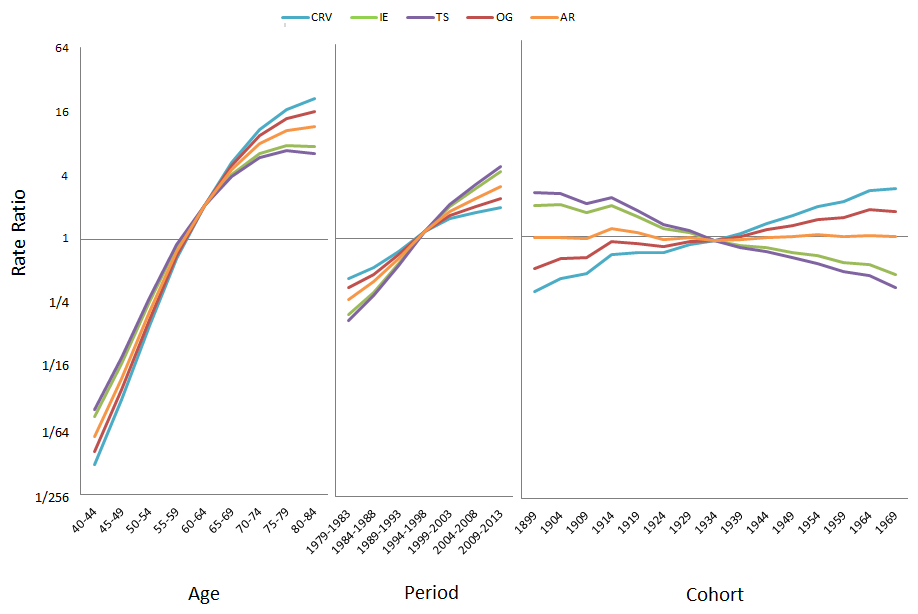

Supplement: S2 Fig — (TIF) [file pone.0226678.s008.tif]
