# Supplementary material for: Age-period-cohort analysis with a constant-relative-variation constraint for an apportionment of period and cohort slopes
Source: PLoS One. 2019 Dec 19;14(12):e0226678. doi: 10.1371/journal.pone.0226678 (PMC6922428; doi:10.1371/journal.pone.0226678)
Supplement: S3 Table — (DOCX) [file pone.0226678.s011.docx]

**Table S3. Monte-Carlo standard error for TS estimates.**

Scenario 1: (A, P_zero_, C_zero_); Scenario 2: (A, P_zero_, C_I_); Scenario 3: (A, P_zero_, C_II_); Scenario 4: (A, P_I_, C_zero_); Scenario 5: (A, P_II_, C_zero_); Scenario 6: (A, P_I_, C_I_); Scenario 7: (A, P_I_, C_I_); Scenario 8: (A, P_I_, C_I_); Scenario 9: (A, P_I_, C_I_); Scenario 10: (i); Scenario 11: (ii); Scenario 12: (iii); Scenario 13: (iv); Scenario 14: (v); Scenario 15: (vi); Scenario 16: (vii).
