# Supplementary material for: Age-period-cohort analysis with a constant-relative-variation constraint for an apportionment of period and cohort slopes
Source: PLoS One. 2019 Dec 19;14(12):e0226678. doi: 10.1371/journal.pone.0226678 (PMC6922428; doi:10.1371/journal.pone.0226678)
Supplement: S6 Table — (DOCX) [file pone.0226678.s014.docx]

**Table S6.** **Prostate cancer incidence rates (per 100,000) in white by age and period groups.**

|  | 1973-1977 | 1978-1982 | 1983-1987 | 1988-1992 | 1993-1997 | 1998-2002 | 2003-2007 | 2008-2012 |
| --- | --- | --- | --- | --- | --- | --- | --- | --- |
| 40-44 | 0.8 | 1.7 | 1.6 | 2.0 | 3.8 | 7.2 | 9.0 | 8.7 |
| 45-49 | 4.5 | 5.7 | 6.0 | 10.9 | 24.3 | 34.1 | 41.3 | 43.7 |
| 50-54 | 20.9 | 25.0 | 29.9 | 56.7 | 105.8 | 138.7 | 148.2 | 138.8 |
| 55-59 | 66.4 | 76.9 | 94.3 | 164.5 | 276.4 | 344.9 | 338.9 | 299.5 |
| 60-64 | 165.2 | 191.7 | 223.6 | 417.5 | 558.5 | 611.0 | 598.9 | 523.8 |
| 65-69 | 316.9 | 364.7 | 450.9 | 796.5 | 907.2 | 941.5 | 889.9 | 787.8 |
| 70-74 | 514.8 | 599.1 | 706.9 | 1215.5 | 1154.1 | 1101.3 | 992.8 | 840.6 |
| 75-79 | 737.9 | 844.2 | 957.4 | 1442.6 | 1124.5 | 1087.4 | 934.0 | 719.1 |
| 80-84 | 978.1 | 1049.5 | 1143.6 | 1533.3 | 1027.5 | 907.8 | 756.4 | 535.4 |
