# Supplementary material for: Age-period-cohort analysis with a constant-relative-variation constraint for an apportionment of period and cohort slopes
Source: PLoS One. 2019 Dec 19;14(12):e0226678. doi: 10.1371/journal.pone.0226678 (PMC6922428; doi:10.1371/journal.pone.0226678)
Supplement: S7 Table — (DOCX) [file pone.0226678.s015.docx]

**Table S7. Parameter estimates and bootstrapped standard error of CRV estimates.**
