# Supplementary material for: Age-period-cohort analysis with a constant-relative-variation constraint for an apportionment of period and cohort slopes
Source: PLoS One. 2019 Dec 19;14(12):e0226678. doi: 10.1371/journal.pone.0226678 (PMC6922428; doi:10.1371/journal.pone.0226678)
Supplement: S8 Table — (DOCX) [file pone.0226678.s016.docx]

**Table S8.** **The prostate cancer incidence rates (per 100,000) in Taiwan by age and period groups.**

|  | 1979-1983 | 1984-1988 | 1989-1993 | 1994-1998 | 1999-2003 | 2004-2008 | 2009-2013 |
| --- | --- | --- | --- | --- | --- | --- | --- |
| 40-44 | 1.5 | 0.8 | 0.8 | 0.8 | 1.1 | 2.1 | 2.6 |
| 45-49 | 2.5 | 2.0 | 2.8 | 4.3 | 4.2 | 6.8 | 10.6 |
| 50-54 | 7.8 | 6.8 | 9.7 | 14.3 | 18.2 | 33.0 | 42.5 |
| 55-59 | 17.3 | 17.6 | 30.7 | 50.7 | 84.3 | 117.5 | 160.0 |
| 60-64 | 44.9 | 54.7 | 82.0 | 144.3 | 219.9 | 307.3 | 413.8 |
| 65-69 | 93.2 | 121.7 | 179.8 | 344.3 | 511.5 | 646.7 | 902.6 |
| 70-74 | 126.6 | 233.9 | 385.5 | 579.5 | 923.7 | 1149.0 | 1489.7 |
| 75-79 | 133.9 | 271.1 | 579.6 | 908.6 | 1193.6 | 1657.2 | 1947.8 |
| 80-84 | 185.2 | 400.7 | 489.7 | 1166.3 | 1502.2 | 1735.2 | 2219.7 |
